# Contested Cumulations: Configurations of Cancer Treatments through the Twentieth Century

**DOI:** 10.1353/bhm.2007.0011

**Published:** 2007

**Authors:** John V. Pickstone

**Keywords:** cancer treatments, ways of knowing, specialization, contested cumulations, path dependency, national differences

## Abstract

The treatment of cancer through the twentieth century may be seen as the successive addition of modalities: first surgery; then radiotherapy, especially between the world wars; and then chemotherapy, from the 1960s. This paper explores some of the systematic differences between the modalities, and how these additions were negotiated in different countries, with different long-term consequences for the development of services and specialization. It focuses chiefly on the United Kingdom and the United States, the former exemplifying a centralized health polity, and the latter, liberal markets combined with large and crucial postwar inputs from government. The differences between health polities were especially important for interwar radiotherapy, which in its centralized form appeared as paradigmatic of the analytical/rationalizing mode in modern medicine. Chemotherapy exemplified a more inventive and experimentalist mode that became common after World War II, and that, through the practice of trials, shaped the new subprofession of medical oncology. The interactions of the modalities, at various levels, are modeled as *contested cumulations* showing strong *path dependency*. The paper ends by reviewing the present situation, especially for Britain, and by underlining the relevance of history.

This paper originated in reflections on the present divisions of specialist labor in cancer hospitals and regional cancer centers in Britain—between medical oncologists who practice chemotherapy, and clinical oncologists who practice radiotherapy with chemotherapy (and who used to be called radiotherapists). This division of labor is similar to that in Swedish cancer hospitals, but substantially different from most of Europe where the doctors who give radiotherapy do not also give chemotherapy, and especially from the United States where medical oncology is a common specialty in community hospitals and office practice as well as in cancer centers and cancer hospitals. And of course, there is a third major party: the surgeons, again differing in organization and their means of advancing practice. The three modalities developed successively, through a long series of professional negotiations; the pathways and the results differ between countries.

Here, then, was an opportunity to see how new modalities had been added to old, in contested cumulations, and how different national patterns could be explained in part through path dependency. But it was also a chance to explore the different structures of practice, knowledge, and politics that characterized each of those modalities, and that may characterize aspects of scientific medicine more generally.^1^

## Modes of Medicine, Contingency, and Path Dependency

In a series of publications, I have tried to develop models of “ways of knowing” and “ways of working” as elements, or ideal types, from which the complex practices of science, technology, and medicine may be said to be compounded. In such configurational models, historical change is usually a matter of contested displacements (rather than successions): [Other P-164] new modes of knowledge and practice come to articulate with previous modes, and these articulations are evident at various scales from the level of “case histories” up to that of “big pictures.” I have argued that *analysis* (with its correlate of rationalized production) and *experimentalism* (correlated with systematic invention) were the modes of knowledge and action most characteristic of science, technology, and medicine over the last two centuries.^2^

Here, I will suggest that radiotherapy, as developed in the centralized, specialist institutions that epitomized the medical modernity of the inter-war decades, was paradigmatically analytical in its forms of work. Radiotherapy institutes included a variety of scientists and technicians as well as doctors; they standardized their therapies and accumulated statistics (about almost all their cases) at a time when most clinicians relied on individual judgments about individual patients. To be sure, cancer chemotherapy, when it developed after World War II, was also highly “scientific” by the standards of contemporary medicine elsewhere; but chemotherapy relied on new kinds of clinical trials—on the experimentalism that had grown from research laboratories, and especially from the testing of new antibacterial remedies. (Indeed, the word “chemotherapy” then covered all these antibacterials as well as the anticancer therapeutics to which the term would later become restricted.) In some senses (for cancer and more generally), experimentalism built on analysis, but the ways of working were different: trials were about using chemical novelties, or inventions, on particular groups of patients. I will suggest that these meta-level differences accentuated the problems of articulation and accommodation when new modalities were added to older ones.

But having sketched the model, and to avoid misunderstandings, I should next mark out the limits of the paper. First, I note that the three therapeutic modalities that are my central concern are but part of the story of cancer treatment, even for recent decades. The analytical-rationalizing and the experimentalist-inventing modes of medicine were always and necessarily accompanied by other ways of knowing—for example, attention to the *natural history* of disease and a concern with *meanings* of the disease to patients as well as to doctors. Practitioners were not just rationalizers or systematic inventors, they also exercised *craft* skills and *rhetorical* devices, even when they were also analyzing and experimenting. [Other P-165] Thus, for example, a fuller account of radiotherapy would include its own symbolic meanings and rhetorical powers; and it would also include the ways in which radiotherapy changed the “natural history” of cancer and its public presence and emotional charge.^3^

Second, I want to clarify my chronological starting point. This is not a full historical narrative of the increasing complexity of cancer therapeutics; for that, we would need to explore how long-standing “bedside” understandings of cancer articulated with mid-nineteenth-century pathological anatomy, and both modes with the new surgery of the late nineteenth century. We would need to explore how some cancers came to be regarded as operable, and how surgical interventions changed customary clinical histories. But in this sketch of twentieth-century cancer therapeutics, I simply take surgery as the first of the main three modalities. In its dependence on the understanding of cancers as local lesions of tissues, modern surgery may be seen as analytical; but the conditions of research and practice in surgery were substantially and persistently different from those in radiotherapy, mainly because, whatever the anatomical models, the industrialization of surgical equipment, or the proliferation of ancillary specialists, the practice of operative surgery has remained essentially craft-based.

And then a third note, to justify the focus on the three main modalities when cancer also involved the many other kinds of medical, nursing, and technical work that have become common in recent medicine—including specialized diagnostic methods and screening, the control of side effects or of pain, physiotherapy, palliative care, psychological treatments, cancer education and prevention.^4^ But here our focus is on the interactions of the destructive measures aimed at the cancer itself, and for (relative) simplicity I largely exclude hormonal treatments and later biological treatments. [Other P-166] Surgery, radiotherapy, and chemotherapy were seen as weapons variously attacking the cancer. One might even take the armamentarium argument further: there is also a characteristic addition *within* some modalities. This is most obvious for recent chemotherapy, which commonly involves cocktails of drugs, and perhaps different cocktails used in sequence.

This cumulation of medical weaponry focused on a single site seems especially characteristic of cancer treatment, and so too are the international differences that have resulted from the differential development of the modalities. Where one mode was developed in a particular way in a particular context, then future “additions” to the system were also likely to be different—not just because the contextual differences may have continued, but because the effects of old contexts had been built into the system.

Yet, as we explore what economists call this “path dependency,” we also need to take account of the countertendencies. Cancer doctors themselves have often been aware of the historical paths and the contingencies built into the treatments they used. And for that reason, from the interwar years, they advocated *teamwork*, in part to try to ensure that the treatment given did not depend on which specialist the patient happened to see first, or which therapeutic specialism was best developed in that particular hospital. Such teamwork, like the multidisciplinary protocols that are now common, may perhaps be seen as a way of limiting history—as producing schedules by which all suitable patients can be treated, whatever their circumstances and wherever they are cared for. Yet, inevitably, to some extent, all such efforts are themselves historically rooted: the forms of teamwork, like the patterns of referral and of treatment, are also shaped by histories.

To be sure, the world of cancer therapy is now global and full of evidence-based protocols that claim international validity. Specialists from many countries attend the big American meetings, and these societies see themselves as international in their reach.^5^ In most countries, the government, the professional communities, and perhaps the medical businesses, produce guidelines on practice which are meant to standardize across their domains. But for all the information now flowing between sites and nations, cancer services and treatments continue to differ between places because of their different histories, and especially the different relative histories of the three professional modalities.[Other P-167]

## The Locations of Cancer Surgery

As Patrice Pinell has well shown for France, it was the development of antiseptic and aseptic surgery that established the curability of some kinds of cancer.^6^ Of course, cancers had been removed before, but at huge immediate risk to the patient. When mortality rates for surgery improved, one could at least assess the proportion of operated cancer cases who survived longer than had been expected without the operation.

Cancer surgery expanded after ca. 1870 both within “general surgery” and in the largely surgical nascent specialties of urology, ENT, and gynecology. Though gastrointestinal cancers remained in general surgery (and stomach cancers remained a major killer through the mid-twentieth century), in all the new specialist fields cancers were a major part of the operative work. For patients in cities, served by specialists, cancers of the cervix and uterus mostly “belonged” to gynecologists, those of the mouth and throat to ENT surgeons, and so forth.

This organizational story of cancer surgery seems simple in outline and fairly similar across most countries. Until recently, few surgeons defined themselves primarily as cancer surgeons—they were “regional” surgeons first and cancer surgeons second. Surgeons usually worked “alone” and did not require hugely expensive equipment. But there are national differences, especially since World War I, partly because surgery then had to articulate with other modalities, notably radiotherapy. In France, Sweden, and Britain, the institutions or hospital departments for cancer that were established or developed between the wars were usually dominated by radiotherapists or pathologists. Most surgery was in general hospitals rather than special cancer centers, unless it was especially complicated and required close association with other specialists.

But in the United States, cancer hospitals, though often started as charities for the incurable, seem to have been dominated from before World War II by surgeons who rode the waves of cancer philanthropy and the powerful cancer-education programs that were characteristic of the United States.^7^ In the 1920s the Memorial cancer hospital in New York was led by a pathologist, James Ewing, and a surgeon, Henry Janeway, who together developed radiotherapy as an independent modality; but by World War II the use of radium and therapeutic X rays had become ancillary either to surgery or to diagnostic radiology. After World War II, [Other P-168] the wartime advances in antibiotics, anesthetics, blood transfusions, and other forms of patient support were seen as expanding the scope of cancer surgery—sometimes, again, at the expense of radiotherapy. Barron Lerner has well shown how American cancer treatment in the 1940s and 1950s was epitomized by heroic surgery, not least at the Memorial whose alumni led the American Cancer Society.^8^

One of the latest, and richest, specialist cancer hospitals was the hospital planned in Texas in 1941 as a state cancer hospital and research facility, and drawn to Houston by a matching grant from the legacy of M. D. Anderson.^9^ Gilbert Fletcher, a French émigré, was recruited to create a radiotherapy department, which claimed to follow European practice; but in the eyes of at least one well-placed commentator, the doctors who shaped the M. D. Anderson Hospital (MDA) “ended up imitating the Memorial Hospital of New York in its division of clinical work prior to the indications for treatment”^10^—which suggests that patients were primarily assigned to the surgeons. Certainly the director and surgeon-in-chief, R. Lee Clark, was a national figure in the American College of Surgeons, especially in its efforts to define the requirements for hospital cancer centers.^11^ The MDA could have chosen another method, which they had in fact observed closely at the Ellis Fischel Cancer Hospital at Columbia, Missouri, where Juan del Regato, a young Cuban with advanced Parisian training, ran the radiotherapy and had about seventy beds—but that was a state hospital for indigents.^12^

Some postwar surgeons were keen to develop laboratory programs—for example, Owen Wangensteen with the physiologist Maurice B. Visscher at Minneapolis.^13^ But generally, if we may borrow the industrial model of research and development (R&D), surgical analyses and technical novelties were more like D than R; they were not conspicuous as discoveries, [Other P-169] or as requiring institutional reconfigurations.^14^ Surgery is still the main treatment for many kinds of cancer, and in the last three decades some surgeons have specialized in cancer; some may be involved in biopsy tests, even for cancers that are then treated by other kinds of specialist. But for all its therapeutic importance, surgery has usually been the dispersed, individualized base on which, or against which, other kinds of cancer specialist would organize. Without ignoring the difference between academic surgery and practice in the medical market, we may refer to the characteristic organization of surgery as “liberal.” Though surgery is based on an analytical understanding of the body, and it long relied on “case series,” its systematic use of experimental statistics dates only from the 1970s; and except perhaps in Soviet Russia, “surgical labor” has proved hard to divide. As we shall now see, in some places, and especially for the surgical insertion of radium, radiotherapeutics followed that liberal model, even when centralized, rationalized facilities were being promoted elsewhere.

## Radiotherapeutics and Surgery: The Liberal Model

Radiotherapy seems to be the modality in which national differences became most apparent and institutionalized; indeed, it is the modality that has been most definitive of *cancer* services in Britain and Europe. But neither radium nor X-ray therapy was peculiarly identified with cancer until after World War I (except in the sense that they could *cause* it). It is across a range of diseases that we must first explore the relations of the new modality to the old.

It was through actions on the skin that X rays and then radium were chiefly recognized as local medical agents (beyond the visualization role of X rays). Both were usually seen as acting like cauteries or caustics—as “burning” the skin. As such they were conceptually related to surgery, but also to the chemical caustics that had long been used against superficial cancers. Partly because *some* skin cancers proved easily tractable (or, like rodent ulcers, were defined out of “cancer”), they tended to become part of dermatology (and marginal to cancer), along with conditions such as ringworm. At the start of the twentieth century in Manchester, the Cancer Pavilion experimented with X rays and, when disappointed, passed its machine to the skin hospital. After the Great War, when a Radium Institute was established in Manchester, its first radiotherapist had been [Other P-170] a dermatologist, but he began to work chiefly on cancers of the mouth and womb; skin cancers were left to the skin hospital.^15^ Before World War I, and beyond the skin, as it were, X rays and radium were used chiefly for palliation—for shrinking inoperable tumors and relieving pain. We tend to forget this “biographical” dimension, but it radically changed the “natural history” of cancer and its social meanings. As Caroline Murphy pointed out, the foul, suppurating cancers were now rarely seen—though radiotherapy could also produce disfigurations. Cancer, for the most part, came to be a dissolution of the body.^16^

Surgery was already the established remedy for accessible cancers, and other treatments were now added into the space labeled “inoperable.” They were envisaged as palliative, as a possible means of extending “cautery” beyond the scalpel’s reach, or perhaps as avoiding surgical “mutilation.” The Manchester Radium Institute had been promoted by a prominent ENT surgeon in the hope of extending the range of “operations” for cancer of the mouth and larynx, and avoiding the chopping out of the tongue and other parts; indeed, such procedures were successfully developed in the 1920s and 1930s.^17^ At the Middlesex Hospital in London, the surgeon who helped develop radium for uterine cancer did so on inoperable patients, but it worked well enough that he came to offer “operable” patients a choice of therapy. He had learned his radium treatment from Sweden, and so did the women doctors of the Marie Curie Hospital in London who established radium as the preferred treatment for cervical cancer.^18^ For breast cancer, the addition of radiotherapy to surgery owed something to redefinitions of the surgically acceptable. Geoffrey Keynes, a cultivated British surgeon who opposed the radical mastectomy and encouraged radiotherapy, viewed the Halsted operation as a “horrible mutilation.”^19^

Inasmuch as radium extended the range of surgery, its use could be seen as an extension of the practice of surgeons who specialized in ENT [Other P-171] or gynecology, just as it proved an extension of dermatology. For hospital *in*patients more generally, the surgeons “owned” many of the beds and so “naturally” controlled the treatment. To some extent, that logic of body-site specialization was indeed followed, especially for brachytherapy—treatment by the insertion of radium needles. The radium needles were easily portable, they required surgical insertion, and they were not hopelessly expensive. They could be part of the practice of a specialist surgeon or gynecologist—and so they commonly were, perhaps especially in the United States, where the use of radium was not regulated by the state and where the American Radium Society was dominated by surgeons.^20^

In this liberal model, all significant hospitals had surgical radium-therapy; X-ray therapy was an annex to diagnostic radiology, and radiologists did not control beds. In other countries, including Britain, this liberal model was also used, but the extent of the practice is not well documented. Because it was not specially “organized” or much linked with research, it is not well known; it followed the customary divisions between (internal) medicine and surgery, extending the latter, as it also extended the para-clinical practice of radiology.

## The Centralized Model of Cancer Services

However, radium and intensive X rays were not “ordinary” therapies. Both were dangerous, especially in the hands of doctors with little understanding of radioactivity; and they were very expensive. The price of radium varied considerably over the interwar period, as demand fluctuated or new sources were discovered, but for a cancer hospital to acquire an amount sufficient for curative and palliative treatments required substantial special charity funding and/or government support. Radium was one of the first medical technologies funded by public appeals, and by the 1920s, in some regions and countries, specialists in radiotherapy were developing schemes for centralized treatment and research.

In the centralized model, all radium treatment and most X-ray treatment was to be given in specialist regional or national institutions which might also be responsible for major cancer surgery. In such institutions, pathologists, diagnostic radiologists, radiotherapists, and surgeons would all be experts on *cancer* and its treatment. They would work in teams, along [Other P-172] with physicists and statisticians and special nurses. The institution would have research laboratories and there would be close collaboration between researchers and clinicians. All patients would be treated by agreed regimes and entered into the statistics by which the regimes could be evaluated. Here was a model of medical modernism, based on analysis and rationalized production—though supplemented by individualized care “beyond the cancer,” as it were.^21^ It gained additional plausibility from the widespread interwar concern with cancer mortality. As deaths from acute infectious disease continued to decline, cancer became a major public health disease, alongside tuberculosis, and cancer “prevention” came to mean early treatment in the hope of preventing death.

In no country were all the elements of the centralized model realized together, but they remained an ideal to which hospital service planners continually had reference, through to the present—for example, the current plan for improving cancer services in Britain.^22^ I do not mean to suggest that planners deliberately went back to old plans; or that they were always aware of the models as realized in key institutions, though this would usually be the case. Rather, the stress on centralization, a multiplicity of special skills, teamwork, and statistics was a model that came naturally to experts in institutions throughout the twentieth century, whether the object for reform was a hospital service, a factory, a research laboratory, or a city government. It was a very general model based on analysis and rationalization.

### France and Sweden as Models

The nearest approach on paper was the plans in France, after the Great War, for regional cancer centers that would include surgery. These, as Pinell has shown, derived from wartime medical organization and from the direct links between wartime organizers and clinicians, scientists, and social elites; they were partly financed by charity, especially initially, but most of the support came from the state. The high profile of cancer arose in part from the public health perception of increasing incidence, in part from French pride in radium and the Curies, and from a link with that other temple of Gallic scientific medicine—the Pasteur Institute; in part, too, from the perception that other countries were getting ahead of [Other P-173] France in the use of radium. This was a project for “scientific medicine,” drawing on tuberculosis services, but ultrascientific in its elaboration. The real developments in France took a different path, however—not (just) because plans of that scale and professional complexity commonly tend to be reduced and compromised in practice, but because there was a popular magic about radium that outran both the laboratories and the planners. In France as in Britain, public charities to provide radium cures had appeared in small towns as well as large cities. If local surgeons and radiologists were given free rein and local support, the resultant services would be dispersed beyond the control of the would-be specialist radiotherapists and such surgeons as took radium seriously; it would therefore be better to limit the geographic spread, to allow most cancer surgery to continue locally, and to centralize for radiotherapy (and for closely associated surgery). As Pinell showed, this new logic, now focused on a therapeutic modality rather than “cancer,” was strengthened by the shift to teleradiotherapy, which used huge amounts of radium at a distance from the patient. The cost and dangers here were so high that such treatments had to be centralized.^23^

Where the French were planning a network, the Swedes already had the Radiumhemmet, a more or less national institution established in Stockholm in 1910 by a radiologist working with a surgeon, and with access to private funds. By 1939 the Radiumhemmet had a worldwide reputation for radiation physics, cancer staging, and statistics, as well as for radium treatments. Similar clinics were established in Lund and Gothenburg, and state and local government funding took over from the Swedish Cancer Society. Stockholm was especially famous for its work on gynecological cancers, for which a separate department had been established before World War I. Gynecological oncology appears to have remained a uniquely Swedish specialism, where radiotherapy and surgery are used together, often by the same specialist, but in the context of a radiotherapy center rather than the gynecological wards of a general hospital.^24^

The French and Swedish systems initially benefited from charitable funds, but both had elite political backing and they soon gained substantial state funding. They came to epitomize the public face of cancer, well beyond the nations directly concerned. In 1926 an international conference on cancer held at Lake Mohonk under the auspices of the American Society for the Control of Cancer had called for more public education [Other P-174] and had heard Claudius Regaud extol the French system of centralized care.^25^ In the United States, some state governments invested in radium as part of their public health programs, and interest was also strong in Britain, among public health doctors and the national Medical Research Council (MRC). How, then, did this governmental interest affect cancer services in the two countries that are most central to this paper?

### British Varieties

Britain’s first Radium Institute had been prompted by King Edward VII, who was cured of a rodent ulcer by methods deriving from Paris. Opened in 1911, it was restricted to radium therapy. Patients were referred by doctors or other hospitals, as outpatients; the London Radium Institute radiotherapist decided on the form of the treatment, but the diagnosis and further care of the patient were not his responsibility, and there was little opportunity for follow-up. Essentially, this was philanthropic provision of an expensive treatment in a way that did not disrupt the normal patterns of private and charity medicine; it fitted the liberal model. By contrast, the special cancer hospital in London (the Marsden) developed a famous radiotherapy department, and some of the London teaching hospitals—especially St. Bartholomew’s (Bart’s), the Middlesex, and the Westminster—were noted for both cancer surgery and radiotherapy. In some other teaching hospitals, radiotherapy was marginal to diagnostics and to surgery, and the long-standing rivalries between the charity teaching hospitals meant that effort in London was dispersed.^26^ It was in the dense industrial region of Manchester that the idea of a centralized service came to be most fully realized.

The Manchester Radium Institute started in a similar way to the London Institute, except that its originator was a surgeon on the staff of the teaching hospital and its chief patron was a local brewer rather than a monarch. Money was raised, partly by (novel) newspaper campaigns, and used to provide a service available to most of the hospitals in the Manchester district. The Institute was located in the basement of the new teaching hospital, alongside the diagnostic radiology and medical electricity services; but it was separately staffed, and eventually the radiotherapist gained a few beds so he could have patients of his own. He also served [Other P-175] the cancer hospital on the same site—de facto another department of the teaching hospital.^27^

After much contention at the end of the 1920s, both the Radium Institute and the Christie cancer hospital moved off the main site to cohabit in a fine new building in a southern suburb. At about the same time they jointly appointed a new director: Ralston Paterson, a Scottish-trained radiologist who had worked in North America, including a spell at the Mayo Clinic (which already employed a research-minded radiotherapist in addition to a diagnostic radiologist).^28^ At the new hospital-cum-radium-institute, in a region more populous than Sweden, Paterson was able to develop a service more like Stockholm’s than that in London. The hospital was directly responsible for the patients, all radiotherapists followed the same regimes, statistics were built up, a research laboratory was developed, there was a mold workshop, and surgeons visited the hospital for complex mixed treatments (or to repair the effects thereof). Paterson worked with a physicist to develop dose regimes that attained international recognition, especially in the British Empire.

By the late 1930s, the Christie was a world center along with Paris and Stockholm. It was also exemplary as a regional service. Some local towns had bought their own radium, but this was brought under Christie control. In all the local towns, clinics were held by specialists from the Christie, and patients requiring radiotherapy were referred to the regional center. There was fund-raising for the Christie throughout the region, and most local authorities also contributed. In effect it was a regional service that monopolized radium treatment, and it was seen as *the* center for cancer treatment.^29^

The Cancer Act of 1939, which (because of the war) never came into effect, sought to extend similar arrangements to other regions. Some big-city regions with independent cancer hospitals, such as Glasgow, already had arrangements approaching those of the Christie—though there was often friction with elite surgeons in the regional teaching hospital. In some other provincial cities, the service was provided through the main teaching hospital.

The patterns varied, but centralization was gaining by the 1930s, partly because of the power of central government over radium. The Medical Research Council had become responsible for the “war-surplus” radium [Other P-176] after World War I, and initially used it for a radium bomb (teleradiotherapy) at the Middlesex Hospital in London. The MRC was then persuaded to distribute the radium to several centers that had technical expertise but not enough radium of their own. A National Radium Trust and a National Radium Commission were set up by the government in 1929 for the purchase and controlled distribution of radium. They encouraged specialist cancer centers and the differentiation of radiotherapy from radiology.^30^ By the late 1930s in Britain, the subprofession of radiotherapy was well recognized; that its separate development was supported by the leading diagnostic radiologists seems to have contrasted with attitudes in the United States and also in France.^31^

Where radiotherapists controlled beds they could try new treatments, systematize record keeping and follow-up, and extend the uses of radiotherapy. They could include brachytherapy, especially if they had some surgical training. Thus for some conditions radiotherapy became an alternative to surgery, and for these conditions, whether a patient received surgery, radiotherapy, or some combination could depend heavily on the accidents of referral and consultation; at limit, the day on which you attended the hospital might determine whether you saw a surgeon or a radiotherapist, and hence the treatment. Thus, as noted earlier, some surgeons and most radiotherapists came to stress *teamwork*, and some ran joint clinics. For example, the Westminster Hospital was known for its Wednesday clinics where the (Russian-born, European-educated) surgeon Stanford Cade led discussions with other surgeons, radiotherapists, pathologists, physicists, and others. He himself was a national expert in radiotherapy and histopathology as well as surgery; his juniors were more specialized.^32^ At the Christie in Manchester there were daily lunchtime clinics, with the same range of expertise but for different cancer sites each day, depending on which surgical clinics were held on that day.[Other P-177]

### The United States and Socialized Radium

In the United States, by contrast, radiotherapy was rarely separated from diagnosis before the 1960s.^33^ As we have noted for the late 1920s, there were key sites of international importance for radium work, including the Memorial Hospital under James Ewing. The Mayo Clinic had separated radiotherapy from diagnostic radiology, and indeed X-ray therapy from radium therapy;^34^ but this differentiation was not much followed, especially after World War II. From the late 1930s, the Memorial had turned to surgery and biochemistry, a shift that seems to have been very influential.^35^ The few would-be radiotherapists, many of whom were European in origin or education, were limited by the professional associations of general radiology on the one hand, and by the dominance of surgeons on the other; they tended to be isolated, for safety, in the basements of hospitals.

In the United States, as mentioned, radium was not regulated, but it was sometimes supplied by governments—and could have been more so, had the politics of organized medicine been less resistant. The radium used at the Memorial in New York, the Huntingdon in Boston, and the Johns Hopkins in Baltimore was supplied through an agreement with the U.S. Bureau of Mines, brokered by Ewing and Howard Kelly. In 1934, Congress was pressed to accept repayment of ten million dollars of Belgian war debt in the form of radium; the American Radium Society, dominated by surgeons, lobbied *against* the proposal, and “the spectre of a government controlled medical specialty was not raised again until after WWII.”^36^

In the 2001 Stetten lecture, David Cantor has described the radium scheme operated by the National Cancer Institute (NCI) from its foundation in 1937. He sees it as a New Deal public health program, extending the cancer-treatment schemes that had been established in some states and that provided radium treatment for the indigent poor (we [Other P-178] have already noted the Missouri state cancer hospital).^37^ Public provision (and centralization) was advocated by some of the leaders of the vocal American Cancer Society, but it was opposed by the AMA and by American radiologists who saw it as a threat to private practice. In the words of an officer of the American College of Radiology: “Private medical practice has everything to offer that could be offered by a bureaucratic government in a war on cancer.”^38^ In 1940, the doctors lobbied (successfully) against a bill to provide federal health insurance that would have covered “medical services and facilities which had become standardized in their nature but which, because of high cost, were seldom used.”^39^ Concerning radium, as with “socialized medicine” more widely, the doctors had their way—though not without contestation. In Canada, the social democratic government of the province of Saskatchewan led other provinces into a radium scheme, as it did later for provincial health insurance.^40^

Generally, the NCI focused on research rather than treatment; but its radium fund was low-profile after the initial purchase, and the radium was not used to support clinical research or to reorganize existing services.^41^ Indeed, by the time the NCI got under way (and here it contrasts with the MRC earlier in the United Kingdom), cyclotrons were attracting attention as a possible means for treatment, and the United States, through the University of California at Berkeley, was a pioneer in that technology.^42^ But for the most part, American radiotherapy remained an appendage of diagnostic radiology, and American cancer hospitals remained dominated by surgeons.

The British government’s research agency, the MRC, had invested in radiotherapeutics from the 1920s, and it was not much influenced by surgeons or the clinical elites more generally.^43^ In the United States, the [Other P-180] main government investments came *after* World War II, and it was a new form of cancer treatment, chemotherapy, that benefited most.

## Post–World War II Cancer Services in the United Kingdom and United States

The wartime atomic program helped produce isotopes and large-scale generators, both of which were soon adapted to medical uses (partly to demonstrate the peacetime potential of nuclear physics). In the United Kingdom these new tools, plus a higher level of funding for research and the provision of more salaried posts under the new National Health Service (NHS), all boosted radiotherapy—though inasmuch as the NHS generalized such benefits across most of specialist medicine, it reduced radiotherapy’s uniqueness as a scientific and salaried service. The National Radium Commission was abolished, but the radiotherapists remained close to the MRC, and in the 1950s when “chemotherapy” was high-profile for several diseases and clinical trials became the preferred mode of assessment of treatments, the MRC also supported some trials of radiotherapy and surgery.

All teaching hospitals now had salaried professorships of medicine, surgery, and obstetrics and gynecology; these departments were expected to do clinical research, to which the MRC was now more friendly than it had been in the 1920s.^44^ But trials of surgery and radiotherapy generally proved harder to manage than trials of new drugs, in part because they had to build on existing treatments. Lung cancer was then conspicuous, because of its increasing incidence and the emergent interest in smoking as a cause, but systematic trials of radiotherapy on nonoperable cases laboriously confirmed the poor expectations.^45^

The case was different for breast cancer, and that radical mastectomy continued to be questioned in Britain after World War II owed much to the strength of the radiotherapists—especially Robert McWhirter of Edinburgh, who challenged the surgeons in territory they regarded as their own. By introducing the statistical methods that were the mainstay of their own modality, radiotherapists increased the sophistication of surgical case-assessment and results appraisal. (As Lerner notes, Britons and other Europeans were also important for the growth of surgical skepticism [Other P-181] in the United States in the 1950s.)^46^ The British debates between surgeons and radiotherapists were heated, but the latter were better placed than their American counterparts; the MRC trials were “interdisciplinary” and well supplied with independent statistical expertise. Such were the conditions for the postwar “rationalizations” across radiotherapy and surgery, into which cancer chemotherapy was also fitted.

In the United States, by contrast, the new and massive state funding went to research rather than public medical services. New machines were developed, which might have offered a path to independence for radiotherapists, and the heavy involvement of government with physics research and the licensing of isotopes might have offered scope for a large-scale training program to man the new machines—but the radiological profession remained wary of government interference, resisting, for example, the regulation of linear accelerators and other technology. Instead, the numbers of cobalt units and high-voltage units increased rapidly, spreading well outside the main centers, while specialists relied on a handful of training programs, especially in new centers such as Stanford University, the M. D. Anderson in Houston, and the Penrose hospital in Colorado (to which del Regato had moved).^47^

At Stanford, very much a center of postwar technology, Henry Kaplan claimed cures of Hodgkin’s disease by wide-field radiation. At the M. D. Anderson, Gilbert Fletcher, who also worked in diagnostic radiology until the 1960s, applied European methods, especially for the head and neck. Like Juan del Regato at the Penrose, Fletcher was European-trained, as were several of the rare medical physicists—another indication, perhaps, of the field’s relatively low status in the United States.^48^ From the 1950s there were attempts to run clinical trials that included radiotherapy—some involving the NCI, and usually led by practitioners of other modalities—but most of them failed; only after 1967 was there a standing organization for radiotherapy trials. Especially in the United States a subprofession that in prewar Europe had been built around analytical treatments and statistics was slow to adapt to the postwar pattern of experimental trials that had emerged from chemotherapeutic laboratories.^49^ Not until the late 1960s were there enough American trainees to establish a subprofession substantially independent of diagnostic radiology; but from [Other P-182] the 1970s it expanded rapidly, as trial programs became well established, especially for radiotherapy and chemotherapy as adjuncts to surgery, and then as facilities were established outside hospitals, in many cases owned by radiotherapists.^50^

But in some ways, the separate facilities underlined the separation of radiotherapy from the mainline clinical charge of cancer patients. American radiotherapy remained bound to its modality and did not become so formative of *cancer* services, or of the public image of cancer—in part perhaps because it achieved its limited autonomy alongside the rise of chemotherapy. Radiologists of any kind were not prominent in the general politics of medicine; they were, however, much occupied with disputes about conditions of employment, though they were among the best remunerated of American doctors.^51^ In a cross-national survey of attitudes toward advanced and metastatic cases ca. 1990, American radiologists were more likely than Europeans or Canadians to “give hope,” accompanied by relatively intensive radiotherapy; they were less likely to manage the terminal care themselves, and more likely to involve a medical oncologist.^52^

## Post–World War II Chemotherapy

If radiotherapy was most obviously developed in European health systems that were state supported and centralized, chemotherapy was led from America, the land of liberal medicine. Indeed, as we shall see, market medicine probably accounts for its later prominence and professional form—but not for its technical origins. From the start of this new modality, government-funded institutions were central, especially the war-work (including antimalarial and chemical warfare) programs. The three strands of postwar American chemotherapy research grew from research programs on mustard gas (alkylating agents), on nutrition (aminopterin), and on antibiotics (streptomycin). One need hardly stress here the key role of the National Cancer Institute and its clinical program from 1955, the Cancer Chemotherapy National Service Center. This acted like a [Other P-183] pharmaceutical company, testing remedies on standardized mice, and organizing multicenter clinical trials in hospital units where treatments were free and doctors (several of whom were avoiding military service by serving research in Bethesda) were salaried, albeit poorly. Even the drug company most involved initially, Burroughs-Wellcome, was sometimes seen as a research charity.^53^

The cancer drug programs were models of laboratory experimentalism extended into clinical experiments; they variously drew on antibacterial research and therapies, especially those for tuberculosis. The borrowed elements included kinetic models, animal models, additive therapies, large organized programs, and clinical trials as central features of an essentially new form of clinical experimentation.^54^ But the story as commonly told has a twist that is curious in historic context: the prominence of blood and lymph diseases in the new image and practice of cancer.

Leukemias, in some senses, had not been cancers before World War II. They were the preserve of general physicians and especially of hematologists. Since there was little to be done for the patients, they lacked clinical interest—but they were attractive for laboratory-based hematologists: there was a good animal model, blood was easy to handle and observe, there were lots of parameters to measure and cell types to distinguish, and hematology, in the United States, if not in Britain, already spanned laboratory and clinic. The medicines developed in the 1940s and 1950s were not considered curative: they would be given until a relapse occurred, then another would be tried, until no further therapy was possible. Here was therapeutic addition in sequence, mostly for palliation; but some long-term remissions were obtained, as well as a few “cures,” especially with choriocarcinoma and some lymphomas.^55^

These medico-professional conditions were present in most advanced [Other P-184] countries, but in the United States, at the NCI, leukemia was chosen as a major focus for public funding linking clinic and laboratory for cancer. The American initiative was marked by the intensity of funding, by regular experimental trials, and then by the intensification of combination treatments in search of cures rather than simply remissions. Different cytotoxic drugs seemed to have similar effects but different side effects—so using them in parallel might increase their effectiveness. The task force for acute lymphoblastic anemia managed to produce cures in children in the early 1960s, and these successes raised hopes and standards. In the 1960s, doctors who specialized in children’s cancers were formed into networks for trials, and other physicians were enrolled to try various drug combinations on adult cancers. New regimes for cancer had been created outside the old limits of the disease, and by the intensive cumulation of chemicals.

Britain and France had a few key centers that featured in these stories. Jean Bernard was the French prince of hematology;^56^ and in Britain from before World War II, Alexander Haddow at the Chester Beatty Institute had been making and testing chemical remedies, with collaborations in Manchester and at the Marsden Hospital in London.^57^ Various single drugs were developed that increased remissions, but many physicians and most pediatricians were skeptical about the benefits of toxic treatments in extending remissions. In Britain, the American remedies seemed unduly aggressive; to some, they were an example of overprescribing.^58^ But there was also a structural question: in post–World War II Britain, radiotherapists were the “keepers of cancer,” and to the extent that they claimed treatment of lymphomas, say, the freedom accorded to physicians (internists) could be restricted.^59^

In countries where physicians had beds for blood diseases, they were well placed to adopt the leukemia therapies. But the fact that chemotherapy [Other P-185] often required intensive care meant that laboratory-based hematologists were not keen to take leukemia patients; nor were radiotherapists well placed for diseases and types of management that largely fell outside their previous experience and training. Generally speaking, the lymphomas, which already involved surgeons and radiotherapists, were a bridge or boundary across which expertise in leukemias and chemotherapies could be extended to solid cancers. In the United Kingdom from about 1974, a few physicians interested in cancer chemotherapy began to seek recognition as medical oncologists, rather than clinical hematologists or general physicians. They stressed the difficulty of the treatments, but also their length and the integration with general medical care.^60^ In this move, as in their therapies, they were following American examples.

The U.K. radiotherapists immediately felt threatened by this tendency to divide oncology into surgical, medical, and radiation aspects. Radiation oncology, they maintained, was an American, not a British, specialism; their U.K. discipline was “radiotherapy and oncology” (a definition that in turn worried the physicians).^61^ Radiotherapists were focused on collecting statistics to incrementally improve the application of radiotherapy, whether by novel machines, or isotopes, or new routines—but they did not experiment (much) on the basis of laboratory work, nor did they expect breakthroughs. Indeed, they were inclined to see the chemotherapies simply as additions to the armamentarium. For them, trials were mainly analyses of established practices and of plausible variants and additions. Chemotherapy was but another way of interfering with the reproduction of cancer (and of all other fast-dividing cells); it was a further means of palliation, or of marginally improving some (poor) survival rates.^62^ One sees here a key feature of additivity in medicine: where proven remedies exist, however imperfect, they set the conditions against which other remedies must be judged—and this can be very difficult, technically and politically, especially where different modalities are involved.^63^ One of the great advantages of acute leukemias for cancer researchers was the lack of [Other P-186] previously established remedies, and the short time span of clinical trials. For the most part, leukemias were an open field, into which new chemical inventions could be launched.

For would-be chemotherapists, with a history of conquering anemias and bacterial diseases, randomized control trials epitomized a new form of clinical experimentalism. The companies were finding or producing a range of new drugs, which could be variously combined, as for example by Donald Pinkel at Memphis; and chemotherapists were becoming connoisseurs of trials.^64^ And the cancer drug trials were well supported by pharmaceutical companies, which were rapidly expanding their range and their ties with clinicians. The successes with blood and lymph cancers (and choriocarcinoma), and especially with children, attracted much attention; they made central to “cancer” a group of disorders that had been marginal to that field.

In the United States, internists and radiotherapists developed rival treatments for Hodgkin’s disease, but the professional situations were not symmetrical: internists had beds and could act as the doctor in charge; radiotherapists were largely confined to a service role—combined with their service role in diagnostics. The therapeutic returns for most solid cancers were marginal, but the support for the research effort, both from the U.S. government and from industry, was enough to create a new branch of internal medicine called medical oncology. The National Cancer Act of 1971 encouraged the formation of cancer centers in which medical oncologists took most of the nonsurgical cases, calling on radiotherapists as necessary. From the 1970s, multimodal trials became common, and medical oncologists were in a good position to recruit surgeons and radiotherapists. Their position was further secured in the 1980s by a massive increase in the number of drugs to be tested, and by the spread of medical treatments beyond the main centers.^65^

That story of research successes received much publicity, at the time and since. But as Gretchen Krueger is now showing, there is also a story of cancer chemotherapy in “ordinary practice,” especially in the United States.

## Chemotherapy and Liberal Practice

The initiative for the formation of a specialist society, the American Society for Clinical Oncology (ASCO), came from the margins rather than from the NCI or the academic establishments, and it did so when chemotherapy was only just beginning to claim cures.^66^ To understand this, we have to shift our focus away from the state-supported medicine we have discussed for the United Kingdom and for the American NCI programs, and onto the conditions of liberal practice in the rapidly expanding American medical market of the postwar decades.

The development of medical oncology in the United States has to be understood as part of the growth of specialism within internal medicine and the concurrent replacement of family practitioners by internists.^67^ But it also seems to derive from the growth of internal medicine in cancer hospitals and of cancer centers in general hospitals. In the Memorial Hospital from the 1930s, for example, and in the M. D. Anderson through the 1950s, one can follow the ways in which internists claimed places alongside surgeons and radiologists. One claim was for the use of hormone therapies, and then after World War II for the use of radioisotopes, not least for thyroid cancer. Other claims were more generic, including internists staffing the diagnostic clinics that were a key feature of American cancer centers from the 1930s, offering diagnosis for the private patients of local doctors, as well as assigning patients who had been sent to the hospital but not to particular clinicians. As clinical instruments such as the ECG became more common after World War II, they too might be used mainly by internists, often working with surgeons.^68^

For all cancer hospital patients, internists were also on hand for such treatments as were beyond the scope or wishes of the surgeons. After World War II at the Memorial, a research fellow examined all the surgical patients and discovered that, aside from their cancers, more than 60 percent of them were ill enough to need a general medical service.^69^ This may have reflected the increased readiness of surgeons to operate on vulnerable patients and/or their undertaking of more-radical operations; either way, surgical ambition in cancer hospitals probably made more work for physicians. More generally, for diagnosis and for therapy, mutual-referral patterns were part of the economy of increased medical specialization. And where cancers were too advanced for surgical treatment, the hospital internists might take them over, sometimes trying the new chemotherapies, especially when the medicines were so toxic and the patients so ill that they required a great deal of care.

From 1964, a few private physicians who saw an opportunity for specialist business were able to recruit academic leaders and the support of the NCI. They eventually gained recognition as *medical* oncologists from the American Board of Internal Medicine (ABIM)—along with several other new specialisms, but ahead of the hematologists who had strong claims over the blood and lymph cancers, and with whom the medical oncologists then negotiated joint programs. That the ASCO and its journal used the term *clinical* oncology may have reflected a distancing from research establishments, and the aspirations of some of the society’s founders that it would bring together internists, surgeons, and radiotherapists who were interested in chemotherapy.

As a branch of internal medicine, clinical oncology in America proved innovative, popular, and remunerative. Research leaders found a large group of interested clinicians, who in their turn connected to a research field with unprecedented funding and visibility, especially from the early 1970s. By 1980, only seven years after registration had started, there were more than 1,700 medical oncologists registered with the ABIM.^70^ Many of these medical oncologists, including some trialists, were in community practice. In the United States, unlike Britain and France, trial organization did not remain the prerogative of special cancer centers or of teaching hospitals, it permeated all levels of practice (and this is increasingly true of radiological partnerships, involved with adjuvant trials), though [Other P-189] it remained organized from the academic centers. Some chemo-trials are now operated through private medical corporations that supply the therapies and franchise the physicians: US Oncology now accounts for 15 percent of the chemotherapy delivered in the United States.^71^ One might here see the experimentalist mode of practice as having extended from pharmaceutical companies to service companies, and from industrial laboratories to distributed clinics. Trials became part of the market, not just for the drugs, but also for medical care; they became part of the attraction of a physician or a hospital.

## British Medical Oncology and Clinical Oncology in Context

In the United Kingdom, the sustained development came in London, from a few hematologists (who in Britain were pathologists and largely confined to laboratory work) and from physicians who took a particular interest in blood diseases. The Royal Marsden, Bart’s, and the Postgraduate hospital at Hammersmith were the main centers. At the Hammersmith, for example, the hematological pathologist John Dacie collaborated with the physician David Galton, who also worked at the Chester Beatty as part of Haddow’s team. In the sixties there was increasing interest in American therapies (and in the work of Jean Bernard in Paris), and several trials for leukemia were organized through the Medical Research Council, which around 1967 set up a Leukaemia Unit at the Hammersmith. British radiotherapists were sometimes hostile (as indeed were some physicians), but by the later 1960s radiotherapists were collaborating in trials—for example, of bisulphan versus radiotherapy for chronic myeloid leukemia.^72^

At Bart’s, Sir Ronald Bodley Scott and Gordon Hamilton Fairley led the treatment of leukemias, and the extension of chemotherapies to lymphomas and other cancers. The geographic extension of the nascent specialism of medical oncology, and its characteristic trials, was funded by cancer charities and the MRC. In the early 1970s, stimulated by the American Cancer Act of 1971, the MRC and the cancer charities designed a response to complement the lavish American funding, taking advantage of the NHS structures. The Imperial Cancer Research Fund opened the first medical oncology unit, at Bart’s, and four regional units were planned. Manchester in 1973 gained the first provincial chemotherapy research unit, funded by the Cancer Research Campaign at the Christie cancer hospital under the leadership of Professor Derek Crowther, who had trained and worked [Other P-190] at Bart’s. At the Christie, Crowther had one ward (twenty-eight beds) for chemotherapy, and he concentrated initially on lymphomas before spreading outward to hematological malignancies and solid cancers. Apart from one surgical ward, the rest (three hundred beds) were manned by radiotherapists. Adult leukemias were mostly treated at the general teaching hospital, the Manchester Royal Infirmary, and childhood leukemia at the Royal Manchester Children’s Hospital (Pendlebury).^73^

There are several structural points of interest here. First, the origin and growth of a subspecialism on the basis of its research—which appears to be an international characteristic of chemotherapy in rich countries. It was through research funding that consultant positions were initially supported, and NHS funding which now supports most of the posts has continued to focus on the research centers. Second, the restriction of major chemotherapy in Britain to regional centers, at least for prescription (some of the treatment could be carried out in district hospitals). And third, the association of chemotherapy with the drive to centralize the diagnosis and treatment of rare cancers, especially of children. In some provincial regions, including Manchester, the treatment of childhood leukemia was centralized from the early 1970s, and from about that date children’s cancers were largely the preserve of pediatricians.

The key here was the focus on clinical trials and on centralizing patients with relatively rare conditions. There was some published evidence that as long as new routines were followed, patients did as well in hospitals that were not pioneering—but such conservative claims were attacked: who could tolerate the “semi-routinization” of treatments when most of the patients still died? And if new regimes were to be developed with due urgency, then trials must not take too long; this meant that more patients must enter trials, so fewer could be left to “ordinary hospitals.” This logic, plus arguments about the fine balance between overdoses and ineffectual underdoses, ensured the centralization of treatments, even when they did not require expensive apparatus.

The same logic might be seen as preventing the chemotherapeutic regimes’ being administered by “oncologists” whose main training was in radiotherapy. If drugs are always “in development” and trials are expensive and time-consuming, and if the drugs are first used in blood diseases where existing specialists have the laboratory background, then [Other P-191] one has the makings of a new specialism of medical oncology—growing beyond the claims of clinical hematology as the drugs are applied in other conditions. But of course, this nascent specialism had to negotiate its position in a complex medical field where general physicians, hematologists, radiotherapists, as well as some pediatricians and surgeons, also had interests.

In Britain, as in the United States, there were experts who saw cancer treatment as a field in which doctors from surgery, internal medicine, and radiology could come together for common training. In Britain, some wanted to create a common specialism, and the title “clinical oncology” was often used to cover all three modalities, or perhaps as an eclectic alternative to medical oncology as a branch of internal medicine.^74^ During the late 1980s and early 1990s, some eminent chemotherapists and radiotherapists tried very hard to establish a joint Faculty of Clinical Oncology, responsible to both the Royal College of Physicians and the Royal College of Radiologists, but they met opposition—including some of the leaders of their respective colleges.^75^ In the United Kingdom, as in the United States, the convergent option proved a weak position; it was easier to specialize *within* each of the three institutionalized main fields. There is a logic of continued divergence here which proved powerful; in a world divided into medicine and surgery (and radiology), it proved hard to maintain that cancer (or any other focus) might become a field of convergence.

Thus in both countries, chemotherapy became largely identified with medical oncology as a branch of internal medicine, and British radiotherapists struggled to maintain chemotherapy as part of their range (and to resist the possible conversion of empty radiotherapy posts to medical oncology). In the United States, the term “clinical oncology” was appropriated early by specialists in chemotherapy; in the United Kingdom, it was unilaterally appropriated by the radiotherapists in 1990, instead of “radiotherapy and oncology,” which had served as their banner from the 1970s.^76^ Both American chemotherapists and British radiotherapists used “clinical oncology” in the titles of their journals, both of which claimed to represent all clinical approaches to cancer. [Other P-192]

## Comparisons, Prospects, and the Uses of History

In Sweden, it seems, the specialization in oncology, historically based on radiotherapy, *has* been able to incorporate chemotherapy, though there are also physicians who specialize in medical treatments of cancer.^77^ By contrast, in the United States, medical oncology has boomed as a very remunerative branch of internal medicine; the drugs are prescribed more readily than in Europe, and medical oncologists seem to be the lead doctors for many kinds of cancer.^78^ Radiotherapists remain treatment-based specialists, their considerable incomes now exceeded by some medical oncologists; around 1990, in Florida, they had to fight to prevent medical oncologists from setting up freestanding facilities that included radiotherapy.^79^

In Britain, medical oncologists have spread out from the teaching hospitals and children’s hospitals, and as chemotherapy becomes more common, especially as an “adjuvant” therapy, more drugs are given by doctors who are not specifically trained in medical oncology. But, as we have seen, the policy remains to restrict major chemotherapy to the regional cancer centers. There the medical oncologists work alongside the “clinical oncologists,” who give chemotherapy in addition to radiotherapy.

These patterns are not static. In France in the 1980s, the special cancer centers that had been developed in the interwar period found themselves marginalized by the growth of specialist medicine and surgery in the regional teaching hospitals; their counteraction was led by medical oncologists who had trained in the United States, who stressed the common focus on cancer (rather than particular organs), along with multi-disciplinary teams, national coordination of research, and clinical trials for the production of protocols. Their prominence in the elaboration of evidence-based medicine in France resecured their roles at the apices of regional hierarchies and as points of reference for difficult cases.^80^

Even in Sweden, the dominance of the oncology tradition was threatened by proposals to divide up cancer treatment in teaching hospitals that were departmentalized according to body sites (so, for example, physicians and surgeons would collaborate over kidney cancer as they do [Other P-193] over kidney failure, and would call in radiotherapy as needed). A national commission in the early 1970s preserved cancer centers, but they were now to include specialists in internal medicine as well as oncologists; and both general oncologists and gynecological oncologists were to have training in internal medicine as well as radiotherapy.^81^

In Britain, there have been moves to reorganize responsibilities and training in “cancer centers” so that all trainees will learn some radiotherapy and some chemotherapy—to create a single group of oncologists whose further specialization would be primarily by anatomical site (though allowing some specialization in practice between modalities). This would be related to the Swedish model, and it would seem to have a certain economic logic; but the proposal has been on hold since the late 1990s.^82^ Cancer protocols are now required for all major hospitals, under national directions that allow limited local discretion. These are intended to equalize treatment across hospital districts by linking all of them with their regional cancer centers. For most cancers, the protocols require the collaboration of specialists in all three modalities.

Whether and how the British subprofessions reorganize may depend on factors both internal and external to clinical medicine—on the continuations of the structural dynamics of which this whole paper is a preliminary exploration. If, as some British *clinical* oncologists claim, the combinatorial possibilities of intensive chemotherapy are becoming exhausted and “routine treatments” are increasingly detached from research, then the case for a partial merger of the two nonsurgical subprofessions will be strengthened. Chemotherapy trials might come to look more like radiotherapy trials, and combined trials would be the rule—ways of making incremental improvements across the range of available modalities. Experimentalism, one might say, would merge back into extended analysis and rationalization. Indeed, in all leading countries, much of the incremental advance of treatments now depends on the testing of the many possible permutations and varieties of the three main modalities. The ever-shifting variables in these complex configurations of care ensure that the combinations tried are but a fraction of the total possible—so even at this level, judgments must still enter; the rationalization is always imperfect, even before we consider the peculiarities of individual patients.[Other P-194]

Other models of the future are also in play. New waves of drugs, biological as well as chemical, could maintain the dynamism of medical oncology and of its experimental outlook. British clinical oncologists might do more with medicines and less with radiation, and perhaps cancer will come to look more like other areas of medicine, and more like the United States—divided between surgery and internal medicine, with radiotherapy as an add-on (though here one notes that since World War II, repeated predictions of the decline of radiotherapy have accompanied the steady increase of its practice).

But for the continued dynamics of clinical and medical oncologists in Britain, new contextual political factors may be important, including the increased managerialism of governments and the impact of the European Union. The new readiness of the British government to manage issues that would once have been strictly professional is striking. Clinical appraisals, the need to demonstrate competence for new procedures, and new regulations about medical training programs are all well under way. Managerial logic may come to be applied to the historical professional divisions that we have discussed here. These new political contexts, and the subspecialization of all modalities according to the cancer-site, could reinvigorate claims that, as in Sweden, cancer doctors should be expert in all nonsurgical modalities, though perhaps more specialized for research.

Here too, the interactions with Europe may be significant. Both medical oncology and clinical oncology are organized partly through European collaborations and associations, notably the European Society for Medical Oncology. If you check the ESMO website, you will find data on which countries recognize medical oncology: Britain has long recognized it, but Sweden appears to have failed to do so. There is a logic of implied deprivation here that may prove persuasive in attempts to regularize professional roles across the European Union, especially if American models are regarded as helpful. But for Europe, as for Britain, there could be other possibilities based on the integration of competences and new forms of training.

Whatever the developments peculiar to particular nations, we can be fairly sure that the treatments in leading hospitals in all of the richer countries will increasingly be carried out according to protocols for “normal” treatments. In the production of trials and protocols, if not necessarily their execution, specialists from all the modalities will take part, and most patients are likely to receive at least two of the modalities. It remains to be investigated how much the recommendations and the practices continue to differ between and within countries, and how these correlate with the [Other P-195] differences in professional structure and dynamics that I have here submitted to historical analysis, albeit in a preliminary manner.

For the present, I hope I have at least raised some key questions about the cognitive and practical relations between modalities in medicine, about the conditions under which they developed differently, and hence about the path dependency of their interactions. Medical history as a discipline is rarely called on now by medical planners and policy analysts: they prefer economic analyses that are much simpler because they are more exclusive. But it is not these unhistorical forms of economics that explain why American cancer patients now need to see so many cancer specialists concurrently, or why the patterns are different elsewhere. Such explanations require history, albeit of a wider and more comparative form than most historians now produce.

One might reply that what really matters is that treatments be based on the best possible international evidence. Yet here too, as I have argued, full rationalization is impossible because of the incredible complexity of possible combinations of remedies. And even if the recommendations were the same the world over, they would still be “historical” for a reason that even medical historians have rarely explored. In laboratories using animals, possible treatments can be tried in any combinations, but inasmuch as all ethical human trials are constrained not to damage the chances of patients—when compared to presently effective treatments—so clinical trials must depend on the order in which treatments were devised. Even in principle, this most logical form of medical evidence is “path-dependent”—if I may again refer to the minimal tribute that analysts of the present, whether trialists or economists, must pay to history. [Other P-196]

## Footnotes

1
	    Cancer history has benefited of late from a series of excellent review articles that build on each other, and that include treatment as well as research: CantorDavid“Cancer,”Companion Encyclopedia of the History of MedicineBynumW.PorterR.LondonRoutledge199353660 LöwyIlana“The Century of the Transformed Cell,”Science in the Twentieth CenturyKrigeJ.PestreD.AmsterdamHarwood199846178 PinellPatrice“Cancer,”Medicine in the Twentieth CenturyCooterRogerPickstoneJohnAmsterdamHarwood Academic200067186 GaudillièreJean Paul“The Cancer Century,”The Cambridge History of Science6The Biomedical and Earth Sciences since 1800BowlerPeterPickstoneJohn V.New YorkCambridge University Press forthcoming ). For another example of how history may allow doctors to appreciate the contingencies of divisions between specialisms, see BakerJeffrey P.“Historical Adventures in the Newborn Nursery: Forgotten Stories and Syndromes,”Clio in the Clinic: History in Medical PracticeDuffinJacalynNew YorkOxford University Press2005105151079
	

2
	    PickstoneJohn V.Ways of Knowing: A New History of Science, Technology and MedicineManchesterManchester University Press2000ChicagoChicago University Press2001 Pickstone“The Biographical and the Analytical,”Medicine and Change: Innovation, Continuity and RecurrenceLöwyI.ParisLibbey19932348
	    
	

3
	    In Pickstone, *Ways of Knowing* (n. 2), there are historical chapters on the meanings of nature and artifacts, and on natural history as a tradition; both are treated as constitutive of “science, technology, and medicine” generally.
	

4
	    See StevensRosemaryMedical Practice in Modern England: The Impact of Specialization and State MedicineNew HavenYale University Press1966 StevensAmerican Medicine and the Public InterestNew HavenYale University Press1971 . For paramedicals, see LarkinGerry“Health Workers,”Companion to Medicine in the Twentieth CenturyCooterRogerPickstoneJohnLondonRoutledge200353142 . A major work appeared too late to be used here: WeiszGeorgeDivide and Conquer: A Comparative History of Medical SpecializationNew YorkOxford University Press2005 . See also the articles edited by PinellPatrice“La spécialisation de la médécine, XIX–XX siècles,”Actes de la recherche en sciences sociales2005156–57 . For professional jurisdictions more generally, see AbbottAndrewThe System of Professions: An Essay on the Division of Expert LaborChicagoUniversity of Chicago Press1988
	

5
	    See the ASCO and ASTRO Web sites.
	

6
	    PinellPatriceThe Fight against Cancer: France, 1890–1940LondonRoutledge2002
	    
	

7
	    PattersonJames T.The Dread Disease: Cancer and Modern American CultureCambridgeHarvard University Press198713770
	    
	

8
	    LernerBarron H.The Breast Cancer Wars: Hope, Fear, and the Pursuit of a Cure in Twentieth-Century AmericaNew YorkOxford University Press2001699111949674
	    
	

9
	    The First Twenty Years of the University of Texas M. D. Anderson Hospital and Tumor InstituteHoustonUniversity of Texas M. D. Anderson Hospital and Tumor Institute1964 . For information on the MDA, I am also indebted to Helen Valier and Emm Barnes.
	

10
	    del RegatoJuan A.“The Unfolding of Therapeutic Radiology in the United States: A Participant’s View and Autobiographical Essay,”del RegatoRadiation Oncologists: The Unfolding of a Medical SpecialtyFairfax, Va.Radiology Centennial1993197209 on pp. 201–2
	      
	    
	

11
	    See R. Lee Clark papers at the McGovern Archive, Houston, Tex., esp. ser. 4.
	

12
	    Del RegatoRadiation Oncologists 10 2012
	    
	

13
	    See WilsonLeonard G.Medical Revolution in Minnesota: A History of the University of Minnesota Medical SchoolMinneapolisMidiwiwin Press19892201801
	

14
	    For comparisons and relationships of industrial and surgical research, see SchlichThomasSurgery, Science and Industry: A Revolution in Fracture Care, 1950s–1990sBasingstokePalgrave2002
	

15
	    MurphyCaroline“Cancer and Radiotherapy in Britain 1850–1950” Ph.D. diss., University of Manchester, Faculty of Technology 1986326
	    
	

16
	    MurphyCaroline“From Friedenheim to Hospice: A Century of Cancer Hospitals,”The Hospital in HistoryGranshawLindsayPorterRoyLondonRoutledge198922141
	    
	

17
	    MagnelloEileenin association with the Wellcome Unit for the History of Medicine, University of ManchesterA Centenary History of the Christie Hospital, ManchesterManchesterChristie Hospital NHS Trust20032335
	    
	

18
	    See Ornella Moscucci, “The ‘Ineffable Freemasonry of Sex’: Feminist Surgeons and the Establishment of Radiotherapy in Early Twentieth-Century Britain,” in this issue.
	

19
	    LernerBreast Cancer Wars8 3337
	    
	

20
	    BogardusCarl R.“Intersociety, Government, and Economic Relations,”Radiation OncologyGagliardiR.A.WatsonJ.F. vol. 3 of the X-ray Centenary History volumes of the American Radiological Society Fairfax, Va.Radiology Centennial199620129 on p. 218
	      
	    
	

21
	    
	      See PickstoneWays of Knowing 2 18182 AmsterdamskaOlgaHiddingaAnja“The Analytical Body,”CooterPickstoneMedicine 1 41734
	    
	

22
	    A Policy Framework for Commissioning Cancer Services: A Report by the Expert Advisory Group on Cancer to the Chief Medical Officers of England and Wales (known as the Calman Hine report) Department of HealthLondon1995
	  
	

23
	  PinellFight against Cancer 610642
	  
	

24
	  LarssonLars-Gunnar“Organisation of Radiotherapy and Clinical Oncology in Sweden: A Historical Review,”Acta Oncologica199534101115860802510.3109/02841869509127227
	  
	

25
	  The conference is discussed in HayterCharlesAn Element of Hope: Radium and the Response to Cancer in CanadaMontrealMcGill-Queens University Press20058184
	

26
	  Murphy, “Cancer and Radiotherapy” (n. 15), chaps. 4 –5.
	

27
	  MagnelloCentenary History 17 2334
	  
	

28
	  Biography of Paterson in del RegatoRadiation Oncologists 10 15566
	  
	

29
	  MagnelloCentenary History 174970
	  
	

30
	  CantorDavid“The MRC’s Support for Experimental Radiology during the Interwar Years,”Historical Perspectives: Essays on the Role of the MRCAustokerJoanBryderLindaOxfordOxford University Press1989181204
	  
	

31
	  PatersonR.“Radiotherapy 1925–59,”Brit. J. Radiol.1973461687010.1259/0007-1285-46-550-7684585005 Murphy“Cancer and Radiotherapy” 15 7.41
	  
	

32
	  Obituary of Stanford CadeLancet197330274546 . The Wellcome Contemporary Medical Archives Collection, London, includes a useful transcript of its interview with some of Cade’s younger colleagues (from 13 October 1993).
	

33
	  See the X-ray Centenary History volumes of the American Radiological Society; and esp. three chapters in Radiation OncologyGagliardiWatson 20 CoxJames D.“Clinical Practice,” pp. 2141 KnightNancy“Training and Education,” pp. 16584 ; and Bogardus“Intersociety” 20
	

34
	  See del Regato, *Radiation Oncologists* (n. 10), for the collection of biographies, esp. those of Ewing (Memorial Hospital), Paterson (Christie/Holt, Manchester), and Fletcher (MDA); and the biographical notes on Desjardins of the Mayo.
	

35
	  See “Report of a Visit to Canada and the United States by a Delegation from the British Empire Cancer Campaign, July 1948,” pp. 5 and 36, in the F. P. Spears Papers, ser. E, box 14, Wellcome Contemporary Medical Archives, London.
	

36
	  Bogardus, “Intersociety” (n. 20), p. 218.
	

37
	  David Cantor, “Radium and the Origins of the National Cancer Institute,” The DeWitt Stetten Jr. Lecture given at the National Institutes of Health, Bethesda, Maryland, 28 June 2001.
	

38
	  Mac Cahal, executive secretary, to the ARC Board of Chancellors, 19 August 1937, quoted in Bogardus, “Intersociety” (n. 20), p. 215.
	

39
	  Ibid.
	

40
	  For Canada, see HayterElement of Hope 25 MaioniAntoniaParting at the Crossroads: The Emergence of Health Insurance in the United States and CanadaPrincetonPrinceton University Press1998
	

41
	  Cantor, “Radium” (n. 37).
	

42
	  For example, see the Minutes of the National Advisory Cancer Council for its initial meetings on 9 and 27 November and 13 December 1937, U.S. National Archives, Washington, D.C., RG G443, box 6; and the biography of Albert Soiland in del Regato, Radiation Oncologists 10 7786
	

43
	  AustokerJoanA History of the Imperial Cancer Research Fund 1902–1986OxfordOxford University Press19886990
	  
	

44
	  Clinical Research in Britain, 1950–19807Wellcome Witnesses to Twentieth Century MedicineLondonWellcome Centre for the History of Medicine2000
	  
	

45
	  See Carsten Timmermann, “As Depressing As It Was Predictable? Lung Cancer, Clinical Trials, and the Medical Research Council in Postwar Britain,” in this issue.
	

46
	  LernerBreast Cancer Wars 8 93101
	  
	

47
	  See Knight“Training” 33 17682 Cox“Clinical Practice” 332836
	

48
	  See the biographical notes on Kaplan, Fletcher, and del Regato himself in del Regato, Radiation Oncologists 1024623738259
	

49
	  Cox“Clinical Practice” 33 3536
	  
	

50
	  Bogardus“Intersociety” 20 22527 Cox“Clinical Practice” 33 3036 OwenJean B.“Recent Patterns of Growth in Radiation Therapy Facilities in the US: A Patterns of Care Study Report,”Internat. J. Radiat. Oncol. Biol. Phys.1992249838610.1016/0360-3016(92)90483-x1447040
	  
	

51
	  Bogardus“Intersociety” 20 2056
	  
	

52
	  MaherE. J.“Treatment Differences in Advanced and Metastatic Cancer: Differences in Attitude between the USA, Canada and Europe,”Internat. J. Radiat. Oncol. Biol. Phys.1992232394410.1016/0360-3016(92)90568-31572821
	  
	

53
	  For the cancer-screening programs, see BudR.“Strategy in American Cancer Research after World War II: A Case Study,”Soc. Stud. Sci.19788425591161043210.1177/030631277800800402 ZubrodC. G.“Origins and Development of Chemotherapy Research at the National Cancer Institute,”Cancer Treat. Rep.1984689196362871 LaszloJohnThe Cure of Childhood Leukemia: Into the Age of MiraclesNew Brunswick, N.J.Rutgers University Press1996
	

54
	  For experimentalism in biomedicine generally, see LöwyIlana“The Experimental Body,”CooterPickstoneMedicine 1 43550 . For clinical trials as a new form of medicine, see Peter Keating and Alberto Cambrosio, “Cancer Clinical Trials: The Emergence and Development of a New Style of Practice,” in this issue.
	

55
	  For an excellent account of medical oncology in the United States, Britain, and France, see LöwyIlanaBetween Bench and Bedside: Science, Healing, and Interleukin-2 in a Cancer WardBostonHarvard University Press19963683 . See also JonesW. G.“Cancer Chemotherapy,”Cancer Topics and RadiotherapyJoslinC. A. F.LondonPitman19823145
	

56
	  Cristelle Rigal, “Contributions à l’histoire de la recherche médicale: Autour des travaux de Jean Bernard et de ses collaborateurs sur la leucémie aiguë, 1940–1970” (doctoral diss., Université Paris 7—Denis Diderot, 2003).
	

57
	  “Alexander Haddow,”Biog. Mem. Fell. Roy. Soc.19772313391 pp. 153–60 11615731
	  
	

58
	  See TobiasJ. S.“Who Should Treat Cancer,”Lancet1981188486 on p. 885 611230310.1016/s0140-6736(81)92150-4 ReesGareth J. G.“What Is Best for the Patient? A European View,”Cost and Benefit in Cancer CareStollBasilBasingstokeMacmillan198831383435
	

59
	  Tobias“Who Should Treat” (n. 58); Rees, “What Is Best” (n. 58); M. J. Peckham, “Clinical Oncology: The Future of Radiotherapy and Medical Oncology,”Lancet1981188687611230410.1016/s0140-6736(81)92151-6 . Also see the editorial in Lancet198126746116051 ; and David Galton’s comments on a period of hostility from radiotherapists, in ChristieD. A.TanseyE. M.Leukaemia15Wellcome Witnesses to Twentieth Century MedicineLondonWellcome Centre for the History of Medicine at UCL200323
	

60
	  Royal College of Physicians (RCP) Paper (78/8), to the Working Party on Oncology, 1978, Royal College of Physicians Archives, London (hereafter RCPA).
	

61
	  Minutes of the Joint Committee on Medical Oncology, meeting at Glasgow, 9 October 1974, RCPA.
	

62
	  For example: “Unlike radiotherapists, who do not expect exciting overnight improvements, the medical oncologists receive periodical bursts of enthusiasm by the perennial advent of any new *drug du jour*” ( del RegatoJuan A.“One Hundred Years of Radiation Oncology,”Current Radiation Oncology2TobiasJeffrey S.PatrickR. MThomas [LondonArnold1996], pp. 135 on p. 31
	    
	

63
	  See Timmermann, “Depressing” (n. 45).
	

64
	  See the account of Pinkel in Laszlo, *Childhood Leukemia* (n. 53), pp. 31–32, 234–38.
	

65
	  In 1981, it was reported that the United States had 1,700 medical oncologists certified (about 6.8 per million) since the recognition of the specialty in 1974, and that most large hospitals had set up departments. In Britain, “by contrast, departments of radiotherapy (which have historically been related to diagnostic radiology) have usually provided an out-patient service without attempting to supervise more general aspects of cancer care” (Tobias et al., “Who Should Treat” [n. 58], p. 885). In 1985 the ratio of medical oncologists to the population in the United States was about thirty-five times that in Britain (Rees, “What Is Best” [n. 58], p. 34). In 1995 there were 81 British consultants in medical oncology, less than a quarter of the number per population that the United States had supported fifteen years previously. The number in Britain increased more rapidly over the 1990s (on NHS rather than academic research funding) to 163 by 2001 and to 220 by 2006 (about 4 per million population) (Derek Crowther, personal communication).
	

66
	  KruegerGretchen“The Formation of the American Society of Clinical Oncology and the Development of a Medical Specialty, 1964–1973,”Perspect. Biol. & Med.200447537511546717610.1353/pbm.2004.0065
	  
	

67
	  Stevens,Rosemary“The Curious Case of Internal Medicine: Fundamental Ambivalence, Social Success,”’Grand Rounds: One Hundred Years of Internal MedicineMaulitzRussell C.LongDiana E.PhiladelphiaUniversity of Pennsylvania Press198833964
	  
	

68
	  These processes can be followed in the (Annual) Reports of the Memorial Hospital (at the Rockefeller Archive, Tarrytown, N.Y.), and in those of the M. D. Anderson Hospital (in the Hospital Archive, Houston, Tex.).
	

69
	  Memorial Hospital Report, 1947–51, Section on the Department of Medicine, Rockefeller Archive, Tarrytown, N.Y.
	

70
	  MilstedR. A. V.“Cancer Chemotherapy—What Have We Achieved?”Lancet19802134346 on p. 1343 610413810.1016/s0140-6736(80)91795-x
	  
	

71
	  See Keating and Cambrosio, “Cancer Clinical Trials” (n. 54).
	

72
	  ChristieTanseyLeukaemia 59 1214
	  
	

73
	  See n. 65. For comparisons of medical oncology in France, Britain, and the United States, see LöwyBench and Bedside 55 6571 . For British policy I am indebted to Helen Valier, who is investigating the international politics; ZuckermanLordCancer ResearchLondonHMSO1972 ; and, re the Manchester scene, Derek Crowther, personal communication.
	

74
	  The term was used generically in the interdisciplinary Working Party on Medical Oncology in 1978–79 (see Minutes, RCPA); and as an alternative to developing medical oncology: see Minutes of the Joint Committee on Medical Oncology (n. 61).
	

75
	  See Minutes of the College Committee on Medical Oncology, 1986–94, RCPA.
	

76
	  Ibid., 15 June 1990.
	

77
	  Larsson, “Organisation” (n. 24).
	

78
	  LöwyBench and Bedside556571 Tobias“Who Should Treat” (n. 58); Gareth Rees, “What Is Best” 58 34
	  
	

79
	  Bogardus“Intersociety” 20 22527
	  
	

80
	  Patrick Castel and Erhart Friedberg, “Institutional Change as an Interactive Process: The Modernization of the French Cancer Centers” (Paper delivered at Institutions Conference, Stanford University, 26–27 March 2004).
	

81
	  Larsson, “Organisation” (n. 24).
	

82
	  For the United Kingdom, see the Royal College of Physicians and the Royal College of Radiologists, Joint Council for Clinical Oncology, *Report of the Working Party on Training and Education in a New Specialty of Clinical Oncology*, April 1998, RCPA.
	

